# Radiosurgery alone versus radiosurgery plus whole-brain irradiation for very few cerebral metastases from lung cancer

**DOI:** 10.1186/1471-2407-14-931

**Published:** 2014-12-11

**Authors:** Dirk Rades, Stefan Huttenlocher, Dagmar Hornung, Oliver Blanck, Steven E Schild

**Affiliations:** Department of Radiation Oncology, University of Lübeck, Ratzeburger Allee 160, 23538 Lübeck, Germany; Department of Radiation Oncology, University Medical Center Eppendorf, Hamburg, Germany; CyberKnife Centre Northern Germany, Güstrow, Germany; Department of Radiation Oncology, Mayo Clinic, Scottsdale, AZ USA

**Keywords:** Lung cancer, Cerebral metastasis, Radiosurgery alone, Whole-brain irradiation, Outcomes

## Abstract

**Background:**

It is unclear whether patients with few cerebral metastases benefit from whole-brain irradiation added to radiosurgery. Since primary tumors disseminating to the brain show different behavior, this question should be answered separately for each tumor type. This study compared both treatments in patients with 1-3 cerebral metastases from lung cancer.

**Methods:**

Ninety-eight patients receiving radiosurgery alone were retrospectively compared to 50 patients receiving radiosurgery plus whole-brain irradiation for local control, distant cerebral control and overall survival. Ten other characteristics were additionally considered including radiosurgery dose, age, gender, Eastern Cooperative Oncology Group (ECOG) performance score, histology, number of cerebral metastases, maximum diameter of all cerebral metastases, site of cerebral metastases, extra-cerebral metastases, and interval from lung cancer diagnosis to irradiation.

**Results:**

The treatment approach had no significant impact on local control (*p* = 0.61). On multivariate analysis of local control, ECOG performance score was significant (risk ratio [RR]: 2.10; *p* < 0.001). The multivariate analysis of distant brain control revealed significant positive associations with radiosurgery plus whole-brain irradiation (RR: 4.67; *p* < 0.001) and one cerebral metastasis (RR: 2.62; *p* < 0.001). Treatment approach was not significantly associated with overall survival (*p* = 0.32). On multivariate analysis, significant associations with overall survival were found for maximum diameter of all cerebral metastases (RR: 1.81; p = 0.008), extra-cerebral metastases (RR: 2.98; p < 0.001), and interval from lung cancer diagnosis to irradiation (RR: 1.19; p < 0.001).

**Conclusion:**

Addition of whole-brain irradiation to radiosurgery significantly improved distant brain control in patients with few cerebral metastases from lung cancer. This improvement did not translate into better overall survival.

## Background

About 50% of all patients presenting with cerebral metastasis from a solid tumor have lung cancer [1]. Therefore, this group of patients requires particular attention. Patients presenting with multiple metastases are generally treated with whole-brain irradiation alone [2]. In contrast, many patients with very few cerebral metastases are considered candidates for local therapies such as neurosurgery and radiosurgery [3, 4]. Whereas neurosurgical resection is generally limited to patients with a single lesion, radiosurgery is also administered for more than one metastasis. If radiosurgery is given, it is still not clear whether it should be supplemented by whole-brain irradiation. Those physicians who hesitate in adding whole-brain irradiation are concerned about the potential for increased risk of neuro-cognitive deficits, which has been shown in a small randomized trial [5]. They argue that in case of new cerebral metastases, additional radiosurgery could be applied. Furthermore, randomized trials revealed that the addition of whole-brain irradiation did not lead to improved overall survival [6, 7]. The protagonists of additional whole-brain radiotherapy argue that the combined approach results in significantly better distant cerebral control and overall brain control [8, 9]. Intracerebral recurrence is an important cause of neuro-cognitive decline and should, therefore, be avoided as long as possible. These randomized trials included patients with cerebral metastases from a great variety of primary tumors [5–9]. However, each primary tumor such as lung cancer is characterized by specific biological and clinical aspects and prognostic factors [10–12]. Therefore, it appears helpful to take a specific look at each primary tumor disseminating to the brain. An important question is, “whether it will be possible to identify primary tumors that require the addition of whole-brain irradiation to radiosurgery in order to improve the patients’ prognoses in terms of tumor control within the brain and overall survival?” The present study aims to contribute to this more detailed approach by comparing radiosurgery alone to radiosurgery plus whole-brain irradiation specifically in lung cancer patients with very few cerebral metastases.

## Methods

### Patients and treatments

Of a cohort of 148 patients who received radiosurgery for 1-3 newly diagnosed brain metastases from lung cancer, 98 patients treated with radiosurgery alone were retrospectively compared to those 50 patients treated with radiosurgery plus whole-brain irradiation. Investigated endpoints were local control of the treated cerebral metastases, distant cerebral control (freedom from new cerebral metastases) and overall survival. Ninety-three of 98 patients (95%) and 47 of 50 patients (94%), respectively, received linear accelerator-based radiosurgery, and the remaining patients received CyberKnife treatment. The selection of the treatment approach for an individual patient was based on the treating physicians’ opinions.

In addition to the treatment approach, ten other characteristics were considered such as radiosurgery dose (<20 Gy *vs*. ≥20 Gy, prescribed to the margin of the metastatic lesions representing the 75-90% isodose level), age at the start of radiotherapy (≤59 *vs*. ≥60 years, median: 59 years), gender, Eastern Cooperative Oncology Group (ECOG) performance score (0-1 *vs*. 2), histology of the primary tumor (adenocarcinoma vs. other histologies), number of cerebral metastases (1 *vs*. 2-3), maximum diameter of all cerebral metastases (≤18 mm *vs*. ≥19 mm, median: 18 mm), main site of cerebral metastases (supratentorial *vs*. infratentorial), extra-cerebral metastases (no *vs*. yes) and the interval from lung cancer diagnosis to irradiation (<12 *vs*. ≥12 months). The distribution of these ten characteristics was not significantly different in both treatment groups (Table 1). This retrospective study has been approved by the local ethics committee (University of Lübeck).

**Table 1:** **Patient characteristics of both treatment groups**

	Radiosurgery alone N patients (%)	Radiosurgery + WBRT N patients (%)	***P***-value
**Radiosurgery dose**			
<20 Gy (n = 63)	38 (39)	25 (50)	
≥20 Gy (n = 85)	60 (61)	25 (50)	0.46
**Age**			
≤59 years (n = 76)	49 (50)	27 (54)	
≥60 years (n = 72)	49 (50)	23 (46)	0.86
**Gender**			
Female (n = 68)	47 (48)	21 (42)	
Male (n = 80)	51 (52)	29 (58)	0.73
**ECOG Performance Score**			
0-1 (n = 93)	64 (65)	29 (58)	
2 (n = 55)	34 (35)	21 (42)	0.68
**Histology**			
Adenocarcinoma (n = 92)	62 (63)	30 (60)	
Others (n = 56)	36 (37)	20 (40)	0.89
**Number of cerebral metastases**			
1 (n = 90)	61 (62)	29 (58)	
2-3 (n = 58)	37 (38)	21 (42)	0.84
**Maximum diameter of all cerebral metastases**			
≤18 mm (n = 75)	50 (51)	25 (50)	
≥19 mm (n = 73)	48 (49)	25 (50)	0.97
**Main site of cerebral metastases**			
Supratentorial (n = 127)	84 (86)	43 (86)	
Infratentorial (n = 21)	14 (14)	7 (14)	0.99
**Extra**-**cerebral metastases**			
No (n = 95)	64 (65)	31 (62)	
Yes (n = 53)	34 (35)	19 (38)	0.89
**Interval from lung cancer diagnosis to irradiation**			
≤11 months (n = 77)	51 (52)	26 (52)	
≥12 months (n = 71)	47 (48)	24 (48)	0.99

### Statistical considerations

The comparison of both treatment groups for the distribution of the ten additionally investigated characteristics was performed with the Chi-square test. The Kaplan-Meier method and the log-rank test were used for the univariate analyses of local control of the treated cerebral metastases, distant cerebral control and overall survival. Those characteristics that were significant or showed a trend in the univariate analysis (*p* ≤ 0.060) were additionally analyzed in a multivariate manner with the Cox hazards proportional model. Patients were followed until death or for a median of 12 months (range: 6-39 months) in survivors.

## Results

Better local control of the treated metastases was significantly associated with an ECOG performance score of 0-1 (*p* < 0.001) and almost significantly associated with a maximum diameter of all cerebral metastases of ≤18 mm (*p* = 0.051) on univariate analyses. The treatment approach had no significant impact on local control (*p* = 0.61). A summary of the results of the univariate analysis of local control is given in Table 2. On multivariate analysis of local control, the ECOG performance score was significant (risk ratio [RR]: 2.10; 95%-confidence interval [CI]: 1.38-3.29; *p* < 0.001), whereas the maximum diameter of all cerebral metastases was not significant (RR: 1.19; 95%-CI: 0.52-2.81; *p* = 0.68).

**Table 2:** **Local control of the treated cerebral metastases (univariate analysis)**

	At 6 months	At 12 months	***P***-value
**Treatment approach**			
Radiosurgery alone (n = 98)	90	80	
Radiosurgery + WBI (n = 50)	98	86	0.61
**Radiosurgery dose**			
<20 Gy (n = 63)	88	76	
≥20 Gy (n = 85)	97	87	0.39
**Age**			
≤59 years (n = 76)	93	89	
≥60 years (n = 72)	93	73	0.50
**Gender**			
Female (n = 68)	95	84	
Male (n = 80)	90	80	0.70
**ECOG Performance Score**			
0-1 (n = 93)	96	88	
2 (n = 55)	86	70	**<** **0.001**
**Histology**			
Adenocarcinoma (n = 92)	95	86	
Others (n = 56)	89	76	0.16
**Number of cerebral metastases**			
1 (n = 90)	93	81	
2-3 (n = 58)	92	84	0.39
**Maximum diameter of all cerebral metastases**			
≤18 mm (n = 75)	91	87	
≥19 mm (n = 73)	95	74	0.051
**Main site of cerebral metastases**			
Supratentorial (n = 127)	93	81	
Infratentorial (n = 21)	88	88	0.20
**Extra**-**cerebral metastases**			
No (n = 95)	94	84	
Yes (n = 53)	90	79	0.10
**Interval from lung cancer diagnosis to irradiation**			
≤11 months (n = 77)	97	83	
≥12 months (n = 71)	89	80	0.46

Significant p-values are given in bold.

**Figure 1:**
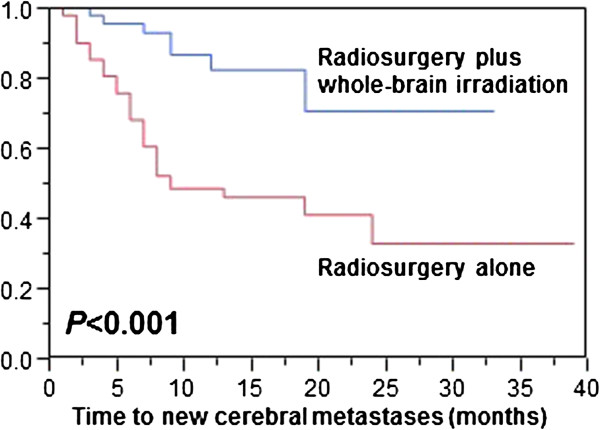
**Comparison of the two treatment groups for distant brain control.**

Distant intra-cerebral control was positively associated with radiosurgery plus whole-brain irradiation (*p* < 0.001, Figure 1) and with presence of only one cerebral lesion (*p* = 0.002) in the univariate analyses (Table 3). In the subsequent multivariate analysis, both treatment approach (RR: 4.67; 95%-CI: 2.23-11.36; *p* < 0.001) and the number of cerebral metastases (RR: 2.62; 95%-CI: 1.49-4.68; *p* < 0.001) remained significant.

**Table 3:** **Distant intra**-**cerebral control** (**univariate analysis**)

	At 6 months	At 12 months	***P***-value
**Treatment approach**			
Radiosurgery alone (n = 98)	68	48	
Radiosurgery + WBI (n = 50)	96	83	**<** **0.001**
**Radiosurgery dose**			
<20 Gy (n = 63)	72	63	
≥20 Gy (n = 85)	82	59	0.92
**Age**			
≤59 years (n = 76)	79	61	
≥60 years (n = 72)	75	61	0.88
**Gender**			
Female (n = 68)	80	57	
Male (n = 80)	75	64	0.52
**ECOG Performance Score**			
0-1 (n = 93)	77	61	
2 (n = 55)	79	61	0.84
**Histology**			
Adenocarcinoma (n = 92)	76	56	
Others (n = 56)	81	71	0.17
**Number of cerebral metastases**			
1 (n = 90)	81	72	
2-3 (n = 58)	73	44	**0.002**
**Maximum diameter of all cerebral metastases**			
≤18 mm (n = 75)	82	63	
≥19 mm (n = 73)	72	59	0.16
**Main site of cerebral metastases**			
Supratentorial (n = 127)	77	59	
Infratentorial (n = 21)	82	72	0.39
**Extra**-**cerebral metastases**			
No (n = 95)	81	63	
Yes (n = 53)	73	57	0.36
**Interval from lung cancer diagnosis to irradiation**			
≤11 months (n = 77)	83	58	
≥12 months (n = 71)	73	62	0.75

Significant p-values are given in bold.

In the univariate analysis of the third evaluated endpoint, overall survival, significant positive associations were observed for an ECOG performance score of 0-1 (*p* < 0.001), a maximum diameter of all cerebral metastases ≤18 mm (*p* = 0.003), and lack of extra-cerebral metastases (*p* < 0.001). A trend towards improved overall survival was observed for an interval from lung cancer diagnosis to irradiation of <12 months (*p* = 0.060). The treatment approach was not significantly associated with overall survival (p = 0.32). The results of the entire univariate analysis are shown in Table 4. In the corresponding multivariate analysis, maximum diameter of all cerebral metastases (RR: 1.81; 95%-CI: 1.1-2.83; *p* = 0.008), extra-cerebral metastases (RR: 2.98; 95%-CI: 1.92-4.64; *p* < 0.001), and interval from lung cancer diagnosis to irradiation (RR: 1.19; 95%-CI: 1.14-1.25; *p* < 0.001) were significant, whereas the ECOG performance score was not (RR: 1.19; 95%-CI: 0.95-1.49; *p* = 0.13).

**Table 4:** **Overall survival (univariate analysis)**

	At 6 months	At 12 months	***P***-value
**Treatment approach**			
Radiosurgery alone (n = 98)	68	49	
Radiosurgery + WBI (n = 50)	78	59	0.32
**Radiosurgery dose**			
<20 Gy (n = 63)	75	51	
≥20 Gy (n = 85)	69	54	0.45
**Age**			
≤59 years (n = 76)	74	63	
≥60 years (n = 72)	69	41	0.10
**Gender**			
Female (n = 68)	82	61	
Male (n = 80)	63	46	0.16
**ECOG Performance Score**			
0-1 (n = 93)	76	61	
2 (n = 55)	64	38	**<0.001**
**Histology**			
Adenocarcinoma (n = 92)	76	64	
Others (n = 56)	64	33	0.10
**Number of cerebral metastases**			
1 (n = 90)	74	54	
2-3 (n = 58)	67	50	0.51
**Maximum diameter of all cerebral metastases**			
≤18 mm (n = 75)	77	62	
≥19 mm (n = 73)	66	42	**0.003**
**Main site of cerebral metastases**			
Supratentorial (n = 127)	72	53	
Infratentorial (n = 21)	67	51	0.72
**Extra**-**cerebral metastases**			
No (n = 95)	86	67	
Yes (n = 53)	45	28	<**0.001**
**Interval from lung cancer diagnosis to irradiation**			
≤11 months (n = 77)	64	47	
≥12 months (n = 71)	80	59	0.060

Significant p-values are given in bold.

## Discussion

For patients with very few cerebral metastases, the benefit of the addition of whole-brain irradiation to radiosurgery is still uncertain. The major concern, which prevents a considerable proportion of radiation oncologists from delivering additional whole-brain irradiation, is the fear of treatment-related decline in neuro-cognitive function. For example, a randomized trial was stopped early, because the rate of neuro-cognitive deficits was significantly greater after radiosurgery plus whole-brain irradiation than after radiosurgery alone at four months following irradiation [5]. However, at one year following treatment, intra-cerebral control was significantly better in the group of patients who had received the combined approach. Unfortunately, the authors did not evaluate the patients’ neuro-cognitive function at one year. This would have been very important because several authors have suggested that an intra-cerebral recurrence of metastatic disease rather than whole-brain irradiation is the major reason of neuro-cognitive deficits in patients irradiated for cerebral metastasis [8, 9]. Therefore, it is very important to prevent such an intra-cerebral recurrence. In the study of Aoyama et al. neuro-cognitive function was better in patients treated with radiosurgery plus whole-brain irradiation when compared to patients treated with radiosurgery alone at one year and two years after treatment [8]. An argument often used against additional whole-brain irradiation is the option of salvage radiosurgery in case of an intra-cerebral recurrence distant from the treated lesions. However, salvage radiosurgery is not always reasonable depending on the number of new cerebral metastases [13]. Therefore, it would be important to have a tool to predict the probability of developing new cerebral metastases. Such a tool has recently been developed [14].

These considerations demonstrate the need for further studies to address the potential benefit of whole-brain-irradiation administered in addition to radiosurgery. As the different primary tumors that lead to cerebral metastases vary considerably with respect to biological and clinical aspects and to prognostic factors, separate analyses of the different tumor types would likely facilitate the identification of patients who may benefit from additional whole-brain irradiation [10–12].

The present study focused on patients with cerebral metastasis from lung cancer, the most common primary tumor among patients with metastases to the brain [1]. According to the results of this study, the addition of whole-brain irradiation resulted in significantly improved distant brain control at six and twelve months following treatment. Given the results of the study of Aoyama et al. improved brain control would likely result in less neuro-cognitive decline [8]. Unfortunately, since the present study had a retrospective design, valid data regarding the patients’ neuro-cognitive function before and after irradiation were not available. This important aspect needs to be investigated in a future prospective trial including only patients with cerebral metastases from lung cancer. Furthermore, the retrospective design of our study should to be taken into account when interpreting the results. Retrospective studies always bear the risk of a hidden selection bias.

In the present study, improvement of distant brain control achieved with the addition of whole-brain irradiation did not translate into improved overall survival. This finding agrees with the results of the available randomized trials of patients with cerebral metastasis from many different primary tumors [6, 7].

These results may lead to the question, whether the addition of whole-brain irradiation is justified if it does not improve overall survival? We feel that it should be offered to most patients with cerebral metastasis from lung cancer, as it can improve distant brain control which likely translates to fewer neuro-cognitive deficits in the long run [8]. One has to be aware that more than half of the patients of the present study developed distant cerebral metastases within one year if treated with radiosurgery alone. Given a more conservative approach, whole-brain irradiation should be offered at least to those patients with a high risk of developing new brain metastases. According to a recent study of patients with cerebral metastases from different primary tumors, those patients with both more than one cerebral lesion and extra-cerebral metastases had the highest risk of developing new cerebral lesions [14]. At least these patients should receive whole-brain irradiation in addition to radiosurgery.

## Conclusions

The addition of whole-brain irradiation to radiosurgery significantly improved distant brain control of patients with very few cerebral metastases from lung cancer. Improvement of distant brain control did not translate into better overall survival in these patients. Further studies with more patients and a longer follow up are required to better define the role of the addition of whole-brain irradiation to radiosurgery in lung cancer patients with very few cerebral metastases.
